# Development and Validation of Prognostic Characteristics Associated With Chromatin Remodeling‐Related Genes in Ovarian Cancer

**DOI:** 10.1002/cam4.70634

**Published:** 2025-02-11

**Authors:** Guansheng Chen, Wenjing Li, Jiayi Guo, Lingyu Liu, Yongjun Wang

**Affiliations:** ^1^ Department of Gynecology and Obstetrics Beijing Jishuitan Hospital, Capital Medical University Beijing China

**Keywords:** chromatin remodeling, immunocorrelation, ovarian cancer, prognostic genes

## Abstract

**Background:**

Ovarian cancer (OC) is a prevalent malignant tumor in the field of gynecology, exhibiting the third highest incidence rate and the highest mortality rate among gynecological tumors. Chromatin remodeling accomplishes specific chromatin condensation at distinct genomic loci and plays an essential role in epigenetic regulation associated with various processes related to cancer development.

**Methods:**

Differentially expressed genes (DEGs) between OC and control samples were screened from The Cancer Genome Atlas (TCGA) and Genotype‐Tissue Expression (GTEx) databases, combined with chromatin remodeling‐related genes (CRRGs) obtained from the GeneCards database to identify differentially expressed CRRGs (DECRRGs). Enrichment analysis and protein–protein interaction (PPI) network were performed on the DECRRGs. Prognostic genes of OC were screened using univariate Cox and least absolute shrinkage and selection operator (Lasso) analyses. A risk model based on prognostic genes was developed, and the survival probability of OC patients in different risk groups was analyzed by Kaplan–Meier (KM) curve. Finally, the expression levels of prognostic genes were validated by quantitative real‐time polymerase chain reaction (qRT‐PCR) and western blotting.

**Results:**

In total, 7 potential prognostic genes associated with the progression of OC patients were obtained, including *ARID1B*, *ATRX*, *CHRAC1*, *HDAC1*, *INO80*, *MBD2*, and *SS18*. Based on the expression level of prognostic genes, OC patients were divided into high‐risk group and low‐risk group. Survival analysis indicated that patients classified into the high‐risk group had higher mortality rates, which enables this prediction model to be utilized as an independent predictor of OC. Immunocorrelation analysis showed that low‐risk patients were more likely to benefit from immunotherapy.

**Conclusion:**

In this study, we have identified 7 prognostic genes, including *ARID1B*, *ATRX*, *CHRAC1*, *HDAC1*, *INO80*, *MBD2*, and *SS18*. Overall, our findings provided a foundation for further comprehension of the potential molecular mechanisms underlying OC pathogenesis and progression.

## 1 Introduction

Ovarian cancer (OC) ranks as the second leading cause of gynecologic malignancy‐related mortality globally. It was estimated that over 239,000 women were diagnosed with OC annually worldwide, resulting in approximately 152,000 deaths [1, 2]. Due to its asymptomatic early stages and insidious onset, coupled with a lack of effective early diagnostic methods, 70% of patients were diagnosed at an advanced stage [3], significantly complicating treatment and prognosis. Over the past three decades, the standard treatment approach for OC has been optimal cytoreductive surgery (R0) combined with chemotherapy (taxane‐platinum regimens). However, despite advancements in surgical optimization and chemotherapy protocols, 75% of women with OC experience recurrence within 3 years of diagnosis [4], and it has a high recurrence rate due to the occurrence of chemotherapy resistance [5]. High‐grade serous ovarian carcinoma (HGSOC) is the most prevalent epithelial subtype, accounting for approximately 75% of cases, and it exhibits a highly aggressive nature with a tendency to develop resistance to chemotherapy early on [6]. Immunotherapy for HGSOC has not yet met expectations, despite the possibility that it may be responsive due to endogenous immunity at the molecular or T cell level [7]. Poly(ADP‐ribose) polymerase (PARP) inhibitors, low‐dose radiotherapy, epigenetic drugs, and anti‐angiogenesis therapy are among the tools available to restore T cell infiltration in HGSOC tumors and could be implemented in combination with vaccines and redirected T cells [7]. Therefore, immunotherapy for OC still needs to be further explored in future studies.

Chromatin remodeling refers to the final step of epigenetic regulation involving processes such as histone modification, DNA methylation, chromatin remodeling, and non‐coding RNA, crucial for determining cell lineage during development [8, 9]. Alterations in this programming facilitates the initiation of embryonic tumorigenesis. Chromatin remodeling establishes specific chromatin condensation states, thereby influencing the activity of chromatin remodelers at specific genomic loci. Chromatin remodeling‐related genes (CRRGs) play pivotal roles in OC, indicating their significant involvement in tumorigenesis [10]. These genes are instrumental in modulating chromatin structure and gene expression patterns, thereby influencing key cellular processes implicated in cancer development and progression [10, 11, 12]. Some forms of OC and other gynecologic diseases have been associated with mutations in specific CRRGs [10]. In summary, chromatin remodeling processes are essential for epigenetic regulation and are intricately linked to the pathogenesis of OC. Further exploration of chromatin remodeling‐related mechanisms promises to uncover new avenues for therapeutic intervention in OC.

In this study, we conducted bioinformatics analysis to investigate 7 prognostic genes associated with OC and chromatin remodeling, including *ARID1B*, *ATRX*, *CHRAC1*, *HDAC1*, *INO80*, *MBD2*, and *SS18*. Subsequently, immune infiltration analysis and drug prediction of prognostic genes were performed, laying the groundwork for comprehending the molecular mechanisms involved in the pathogenesis and progression of OC and chromatin remodeling.

## 2 Materials and Methods

### 2.1 Data Collection

The TCGA‐OC (https://portal.gdc.cancer.gov/repository) dataset encompasses sequencing data from 379 OC patients, whereas the GTEx (https://xenabrowser.net) dataset comprises data from 88 individuals with normal ovarian function [13]. The two datasets were merged as a training set to create a comprehensive dataset for differential gene screening. Additionally, we acquired the GSE140082 dataset from the GEO database (https://www.ncbi.nlm.nih.gov/geo) as validation, which comprises transcriptome sequencing expression profiles of 380 samples for OC. Finally, a total of 117 chromatin remodeling‐related genes (CRRGs) (Table S1) were retrieved from the GeneCards database (https://www.genecards.org).

### 2.2 Screening of Differentially Expressed CRRGs (DECRRGs) in OC

Using the “limma” package (v 3.54.0) [14], we identified differentially expressed genes (DEGs) between OC and control samples based on the TCGA‐OC dataset and the GTEx sequencing data of normal ovarian tissues. The threshold was |log_2_FC| > 1 and *p* < 0.05 [15, 16, 17, 18]. We generated the heatmap and volcano plot using “pheatmap” (v 1.0.12) [19] and “ggplot2” packages (v 3.4.1) [20], respectively. Then, we intersected CRRGs with DEGs to screen out DECRRGs associated with chromatin remodeling in OC. Finally, we used the “RCircos” package (v 1.2.2) [21] to annotate the genomic locations of DECRRGs.

### 2.3 Enrichment Analysis and Protein–Protein Interaction (PPI) Network Analysis of DECRRGs

The DECRRGs were subjected to Gene Ontology (GO) and Kyoto Encyclopedia of Genes and Genomes (KEGG) functional enrichment analysis using the “clusterProfiler” package (v 4.6.2) [22]. The background gene set for enrichment analysis was CRRG gene set. GO enrichment was analyzed with a *p* < 0.2, and since KEGG was not enriched to the pathway when the *p*‐value threshold was set to 0.2, there was no limited threshold for KEGG analysis. Additionally, the PPI network of DECRRGs was constructed by the STRING database (https://www.string‐db.org/).

### 2.4 Screening and Validation of Prognostic Genes

Based on DECRRGs in TCGA‐OC, we used the univariate cox regression to identify candidate genes with *p* < 0.25. Then, the “glmnet” package (v 4.1.8) [23] was continued to be used to screen prognostic genes by least absolute shrinkage and selection operator (LASSO) regression analysis. The Kaplan–Meier (KM) curve for each prognostic gene was plotted using the “survminer” package (v 0.4.9) [24] to assess its impact on patient survival rate. Additionally, the visualization of relationships between prognostic genes was achieved using the “circlize” package (v 4.0.2) [25]. Furthermore, a co‐expression network comprising the top 20 genes exhibiting high proximity to prognostic genes was constructed utilizing GENEMANIA (http://genemania.org/search/).

### 2.5 Construction of the Risk Model for OC

According to the results of Lasso regression analysis, a risk model incorporating prognostic genes and their coefficients was constructed. The risk scores for all OC samples were calculated using the formula: risk score = 0.25068605392974 × *ARID1B* + 0.248372443944594 × *ATRX* + 0.165430860927395 × *CHRAC1* + 0.0790355908046148 × *HDAC1* + 0.148300115000919 × *INO80* + 0.165492627913707 × *MBD2* + 0.0727287417686984 × *SS1*8. Patients in the high‐risk and low‐risk groups were analyzed in the TCGA‐OC dataset and GSE140082 dataset; their mortality and survival were compared, and heatmaps were drawn. Furthermore, clinical information in the TCGA‐OC dataset was utilized to investigate the clinical characteristics associated with these risk groups.

### 2.6 Validation of the Risk Model

To assess the discriminative ability of the risk model to discriminate OC patients with distinct risks, the “survival” (v 3.5–3) [26] and “survminer” packages (v 0.4.9) [24] were used to perform survival analysis on overall survival (OS), and the KM curves were drawn. The log‐rank test was used to compare the survival differences between high‐risk and low‐risk groups. Additionally, we conducted uniform manifold approximation and projection (UMAP) and the *t*‐distributed stochastic neighbor embedding (tSNE) analyses to explore the ability of risk groups to differentiate patients based on both TCGA‐OC dataset and GSE140082 dataset.

### 2.7 Prognostic Nomogram Based on Risk Score and Clinical Characteristics

To evaluate the ability of the risk model as an independent prognostic factor for OC, univariate and multivariate Cox regression analyses were performed for risk score and clinical characteristics, respectively. Based on the risk score and patient clinical data, a nomogram was constructed through the “rms” package (v 6.8.0) [27]. Calibration curves were drawn to assess the degree of match between the 1‐, 3‐ and 5‐year survival rates predicted by the nomogram model and the actual survival rates.

### 2.8 Enrichment Analysis and Immune Infiltration Analysis

To more fully understand the molecular characteristics and underlying mechanisms between different risk groups of OC, we performed enrichment analyses. Firstly, DEGs in two different risk groups were screened using “limma” package (v 3.54.0) (|log2FC| > 0.5, *p* < 0.05) [14], and a volcano map and heat map were drawn by “ggplot2” package (v 3.4.1) [20] and “pheatmap” package (v 1.0.12) [19] respectively. MSigDB (http://software.broadinstitute.org/gsea/msigdb/index.jsp) was used to obtain the gene set marked by hallmarkers for gene set enrichment analysis (GSEA), and “GSVA” package (v 1.46.0) [28] was used for gene set variation analysis (GSVA). The Single sample gene set enrichment analysis (ssGSEA) algorithm was used to quantify the relative abundance of 28 types of immune cell infiltration in samples from high and low risk groups of OC. Then, a box plot was drawn to compare the differential immune cells in the high and low risk groups. In order to explore the correlation between risk score and differential immune cells, Pearson method was used to calculate the correlation coefficient between differential immune cells and the risk score (*p* < 0.05, |cor| > 0.5), and the correlation lollipop graph was drawn.

### 2.9 Prediction of Immunotherapy Response

Tumor immune dysfunction and exclusion (TIDE) scores were calculated for each OC sample based on the training set. The stromalscore, immunescore, and estimatescore were calculated for the high‐ and low‐risk groups using the TIMER algorithm (http://timer.cistrome.org/). The differential expression of immune checkpoint molecules between high‐ and low‐risk groups was assessed by Wilcoxon analysis. A box plot was drawn using the “ggplot2” package (v 3.4.1) [20] to visualize the expression of immune checkpoint molecules in different risk groups.

### 2.10 Drug Sensitivity Analysis

We conducted a comprehensive analysis of multiple chemotherapeutic agents using the GDSC database (https://www.cancerrxgene.org/) to assess drug sensitivity in two different risk groups. The relationship between drug sensitivity and cell line expression profile was established using a prediction model based on ridge regression. The IC_50_ value differences between the high and low‐risk groups in the TCGA‐OC were analyzed using the Wilcoxon test.

### 2.11 qRT‐PCR

Control cell IOSE‐80 and human OC cell SKOV3 purchased from Wuhan Pricella Biotechnology Co. Ltd. were cultured in the 6‐well plates and placed in the 37°C, 5% CO_2_ incubator. Cell lines IOSE‐80 were cultured in medium containing RPMI 1640, 10% FBS, and 1% PS; cell lines SKOV3 were cultured in medium containing McCoy's 5A, 10% FBS, and 1% PS. If the number of cells was large enough, the RNA was extracted using TRIzol (T9424, Sigma‐Aldrich), with 3 samples in each group. Subsequently, the extracted RNA was converted into complementary DNA (cDNA) according to the reaction system (Table 1) and procedure (37°C 15 min, 85°C 5 s) prescribed by the Thermofisher, N8080234 reverse transcription kit. The products were isolated on 2% agarose gel and developed with GelRed (D0140, Beyotime). qRT‐PCR was performed using Platinum SYBR Green mix (11,744,500, Invitrogen) on LightCycler 480 (Roche). A 2^−ΔΔCt^ method was used to determine gene expression levels. The primers used were listed in Table 2. The reaction procedure was shown in Table 3, and the reaction system was shown in Table 4. Finally, the results of qRT‐PCR were analyzed and visualized by Graphpad Prism 6 and compared between different groups by *T*‐test.

**TABLE 1:** Reaction components of the reverse transcription.

System (total 10 μL)	Volume
Total RNA	500 ng
5 × PrimeScript RT Enzyme Mix	2 μL
DEPC Water	Supplement to 10 μL

**TABLE 2:** The primer sequences of prognostic genes.

Primer sequence	
Primer name	Sequence5’‐3’
H‐GAPDH	F:5‘‐AGTCCACTGGCGTCTTCACC‐3’
	R:5‘‐TGATCTTGAGGCTGTTGTCATACTTC‐3’
H‐ARID1B	F:5’‐ACCACCTCCACCACCACCAC‐3’
	R:5’‐TGCTGCTGCTGCTGCTGTTG‐3’
H‐ATRX	F:5’‐GCCGTGACTCAGATGGAATGGATG‐3’
	R:5’‐TCGACCAAGGTTGCGTAGAATGC‐3’
H‐CHRAC1	F:5’‐CAGGAGGCGTTGGTGCTCAC‐3’
	R:5’‐TTTCCTTTCCACTGCCGTGTCTG‐3’
H‐HDAC1	F:5’‐ACATTGCGGCTCATATCAAGAAGG‐3’
	F:5’‐GCCACAGAACCACCAGTAGACAAC‐3’
H‐INO80	F:5’‐ACCGCAGTGCCATTGGATTCTTAC‐3’
	R:5’‐GCCAGACTCCCTCCTTCCTTCAG‐3’
H‐MBD2	F:5’‐CCACGGAGAGCGGGAAGAGG‐3’
	R:5’‐TGCCAGCACTTAGCCCAGATTTTC‐3’
H‐SS18	F:5’‐CACCTCCACGCTCTCACAACATG‐3’
	R:5’‐CATCTGGCCGTTCATCTGGTTCTG‐3’

**TABLE 3:** Reaction components of qRT‐PCR.

System (total 10 μL)	Volume
cDNA	2 μL
F and R primers	0.4 μL each
2 × SYBR Green Mix	5 μL
Deionized water	2.2 μL

**TABLE 4:** qRT‐PCR reaction procedure.

Cycle number	Reaction temperature	Reaction time
1 cycle	95°C	1 min
	95°C	15
40 cycles	58°C	15
	72°C	30
1 cycle	4°C	∞

### 2.12 Western Blotting

The harvested cells were lysed by thermal denaturation using RIPA cell lysis buffer (Sigma aldrich 20‐188). Subsequently, trypsin (Servicebio, G4004) digestion was performed to obtain cell protein precipitates, which were then collected. Protein lysis was achieved by adding RIPA lysate to the protein sample, and the resulting protein supernatant was collected. The concentration of proteins in the supernatant was determined using the BCA protein assay kit (Thermofisher, A55865) following the provided instructions. After equalization, 10 μg of each protein sample was processed via SDS‐PAGE, transferred to PVDF membranes, and blocked using 5% non‐fat milk (232,100, BD Biosciences) diluted in 1 × Tris‐buffered saline supplemented with 0.5% Tween‐20 (TBST). These membranes were incubated overnight at 4°C with primary antibodies, followed by their HRP‐labeled secondary counterparts (W4011 for rabbit and W4021 for mouse originated primary antibodies, Promega). The immunoreactive bands were visualized by enhanced chemiluminescence (ECL). The CHRAC1 antibody (12126‐2‐AP) was diluted with Antibody Dilution Buffer (P0023A, Beyotime); the ratio of antibody to buffer is 1:500. The secondary antibodies were diluted in 1 × TBST. GAPDH was used as the control. Band quantification was done via ImageJ. The experiment was conducted with biological replicates (*N* = 3).

### 2.13 Statistical Analysis

This study adopted the R package (version 4.2.1) [29] for statistical analysis and used the Wilcoxon test and *t* test to conduct significance tests on the difference between the two groups of samples. When the *p* < 0.05, it was considered statistically significant.

## 3 Results

### 3.1 Identification of Differentially Expressed CRRGs in OC

**FIGURE 1:**
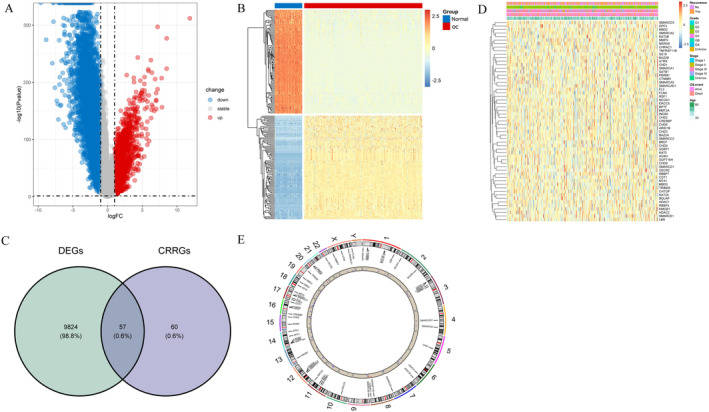
Identification of DEGs (A) Volcano plot of DEGs in OC; (B) heatmap of DEGs in OC; (C) heatmap of 57 DECRRGs; (D) venn map of DEGs associated with chromatin remodeling; (E) chromosome circle diagram of DECRRGs. DECRRGs, differentially expressed chromatin remodeling‐related genes; DEGs, differentially expressed genes; OC: ovarian cancer.

After analyzing the DEGs between tumor and normal samples in the merged dataset of TCGA‐OC and GTEx, a total of 9881 DEGs were identified. Compared with the control, a total of 7759 genes were found to be down‐regulated, while 2122 genes were up‐regulated in OC (Figure 1A). The heatmap of DEGs was shown in Figure 1B. By performing intersection operations between DEGs and CRRGs, we ultimately obtained a set of 57 DECRRGs (Figure 1C, Table S2). The heatmap of DECRRGs was presented in Figure 1D. The circular diagram illustrating the chromosomal distribution of genes was depicted in Figure 1E, with gene loci distributed across all chromosomes.

### 3.2 Analysis of Enrichment of DECRRGs and Construction of PPI Network in OC

**FIGURE 2:**
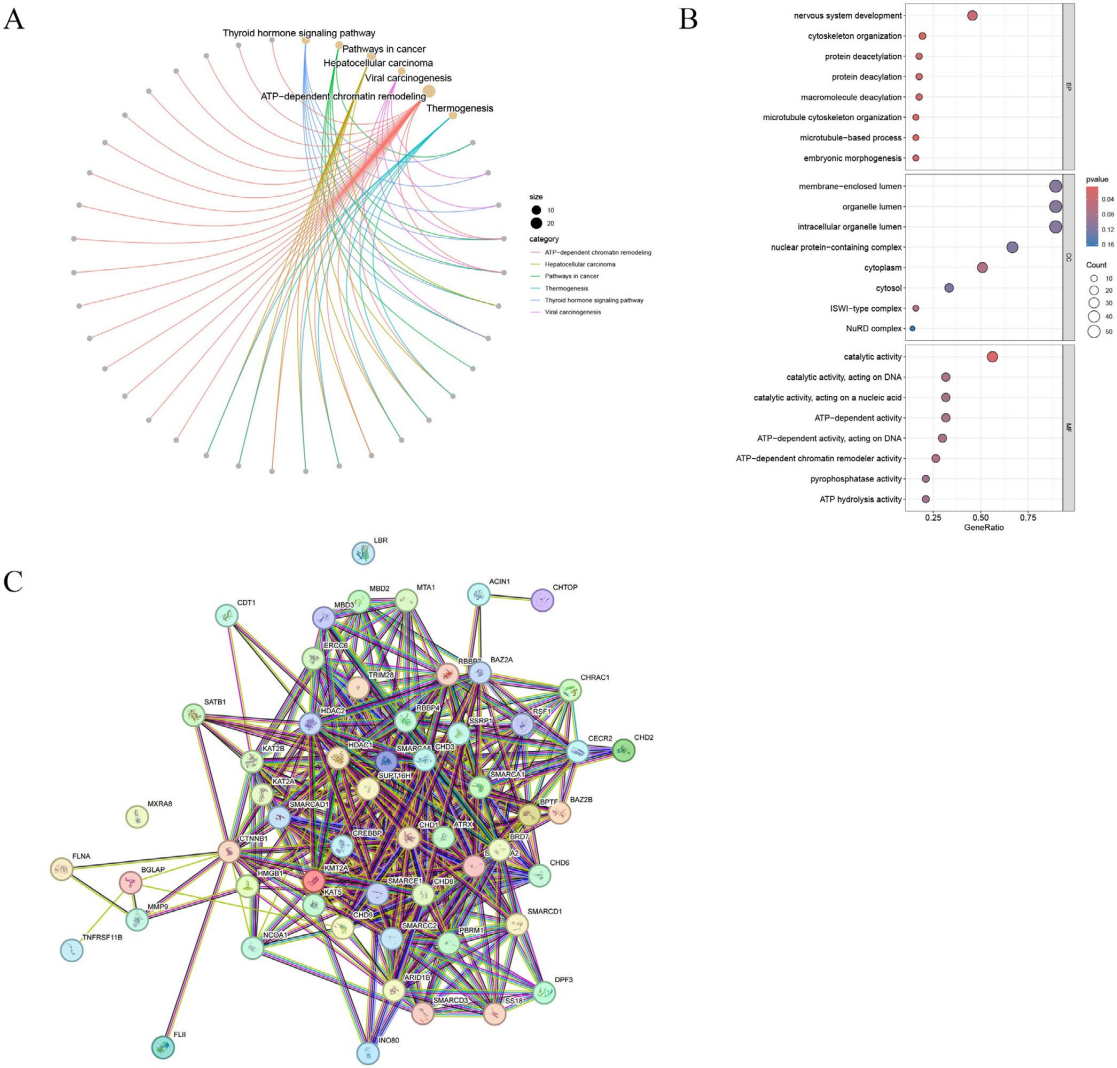
Enrichment analysis of DECRRGs (A) KEGG enrichment analysis; (B) GO enrichment analysis; (C) PPI network of DECRRGs. GO, gene ontology; KEGG, Kyoto Encyclopedia of Genes and Genomes; PPI, protein–protein interaction.

To further understand the molecular mechanisms involved in the development of OC, we performed functional enrichment analysis. The KEGG enrichment analysis demonstrated that the DECRRGs were predominantly enriched in ATP‐dependent chromatin remodeling, pathways in cancer, viral carcinogenesis, and transcriptional misregulation in cancer (Figure 2A, Table S3). The GO enrichment analysis revealed that the significantly associated genes were primarily involved in ATP‐dependent chromatin remodeler activity and positive regulation of DNA‐templated transcription (Figure 2B, Table S4). The PPI networks of DECRRGs were depicted in Figure 2C, illustrating the intricate interconnections among them.

### 3.3 Screening and Validation of Prognostic Genes

**FIGURE 3:**
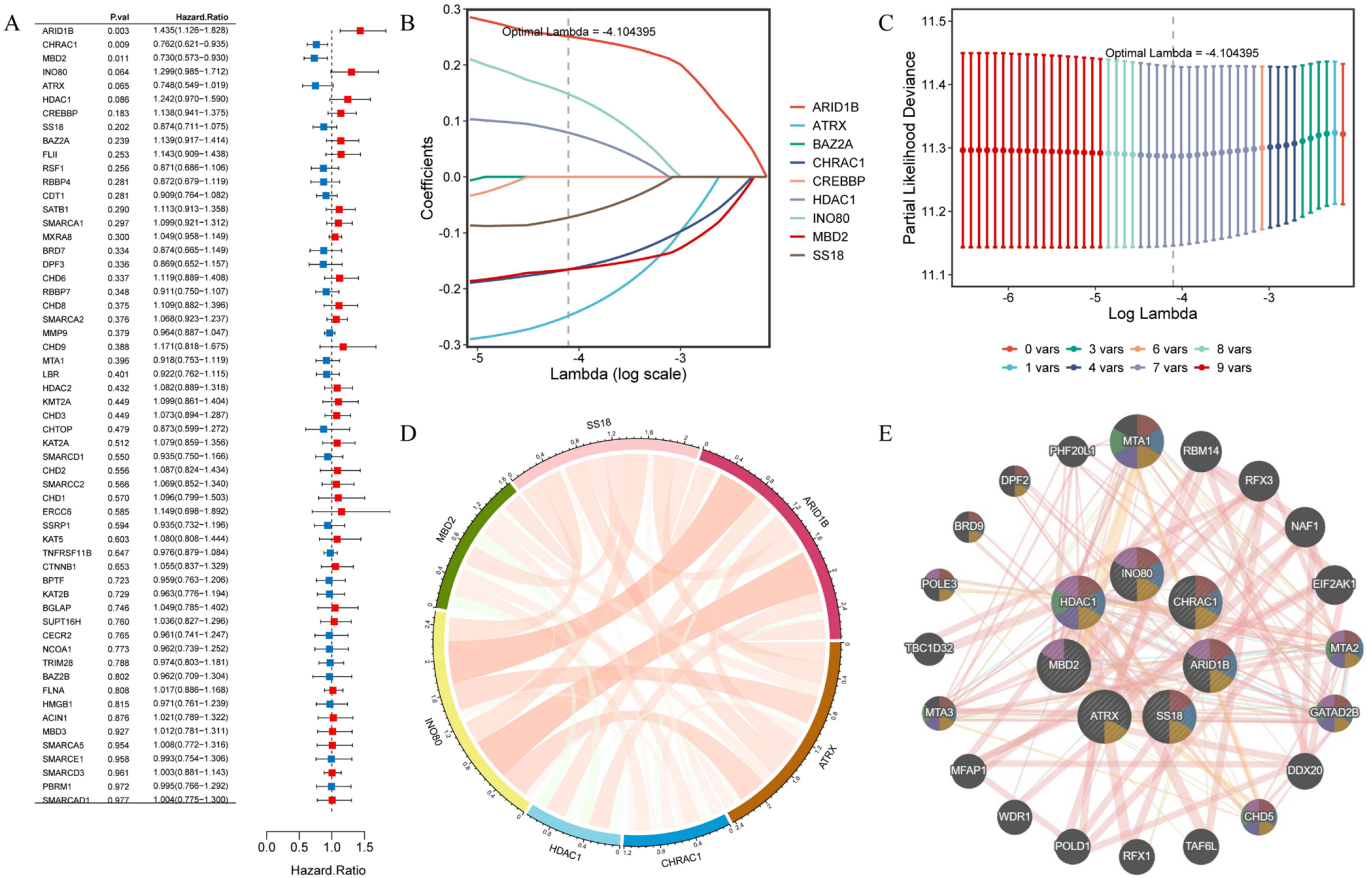
Screening of prognostic genes (A) univariate Cox regression analysis of forest map; (B, C) Regression coefficient path graph and verification curve of LASSO logistic regression algorithm; (D, E) Prognostic gene interaction network diagram. LASSO, least absolute shrinkage and selection operator.

**FIGURE 4:**
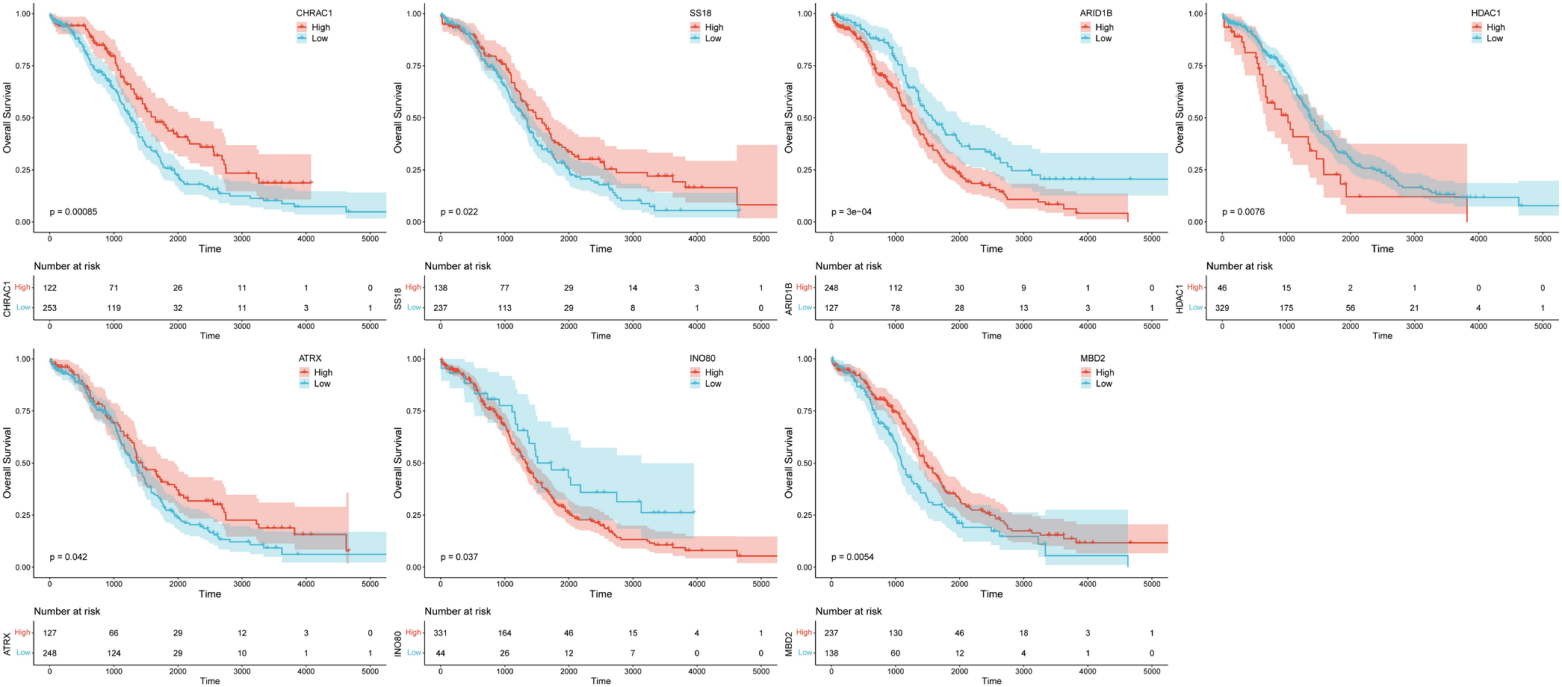
KM curve of prognostic genes. Red curve represented high‐risk group and blue represented low‐risk group. KM, Kaplan–Meier.

To identify genes closely associated with OC prognosis from DECRRGs, prognostic genes were screened. First, 9 candidate genes associated with OC survival were identified by univariate Cox regression analysis (*p* < 0.25) (Figure 3A). Following this, 7 genes with non‐zero coefficients were retained as prognostic genes for OC patients by LASSO analysis (*ARID1B*, *ATRX*, *CHRAC1*, *HDAC1*, *INO80*, *MBD2*, and *SS18*) (Figure 3B,C). The expression of these 7 prognostic genes was analyzed, and the results showed that the expression of CHRAC1 was increased, while the expression levels of the other 6 genes were down‐regulated compared to the control (Table S5). Figure 3D,E showed the correlation analysis of 7 prognostic genes and their interactions with other genes, which were closely related. After conducting survival analysis, we generated KM curves for 7 prognostic genes. The results showed that high expression levels of *ARID1B*, *HDAC1*, and *INO80* were significantly associated with a poorer prognosis. Conversely, elevated expression of *ATRX*, *CHRAC*, *MBD2*, and *SS18* was indicative of a more favorable prognosis (Figure 4).

### 3.4 Survival Analysis of OC Patients in High‐ and Low‐Risk Groups

**FIGURE 5:**
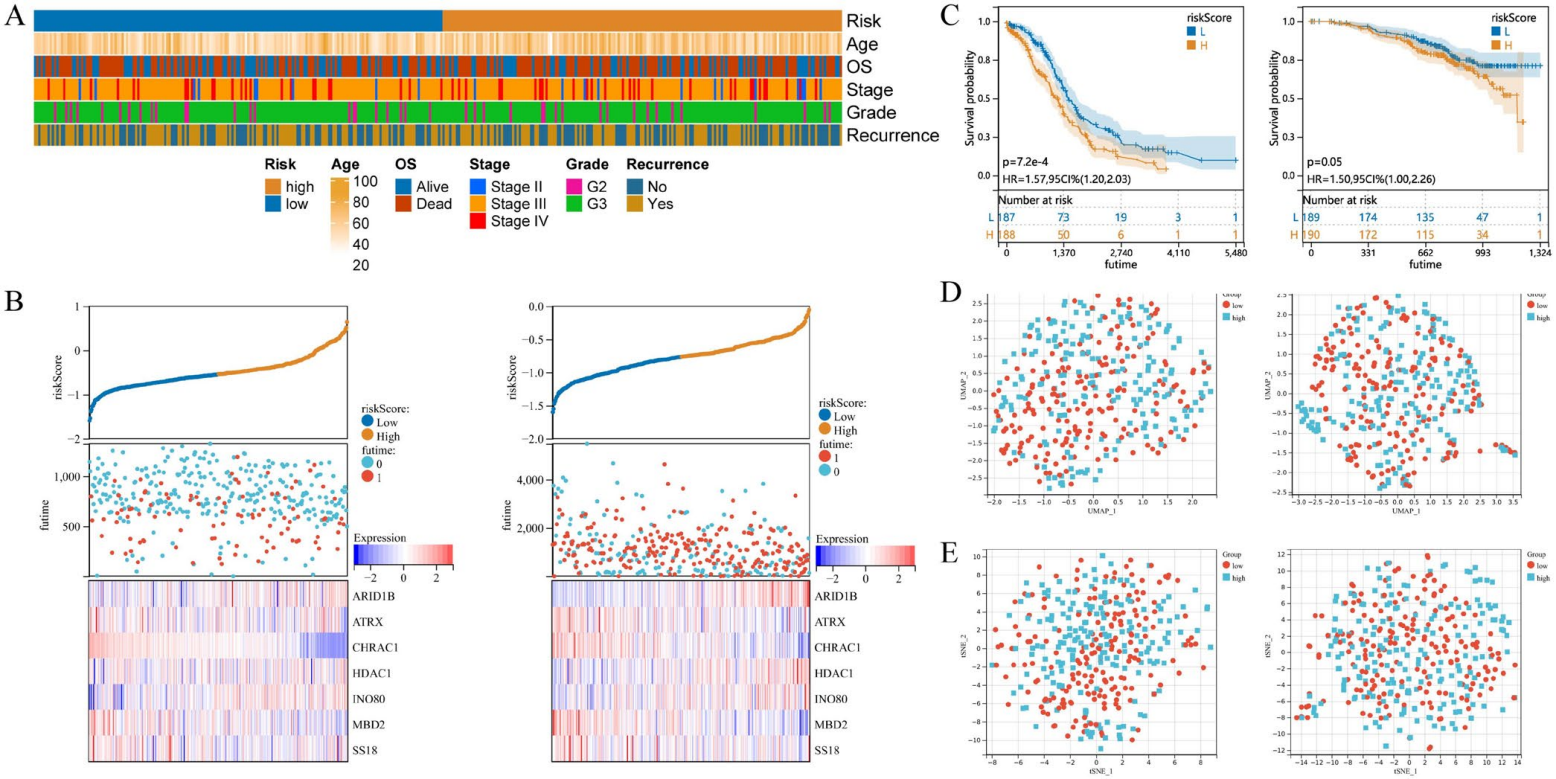
Construction of risk model (A) Clinically relevant heatmap; (B) Triptych of risk scores in the training set and validation set; (C–E) KM curve, UMAP, tSNE in the training set and verification set.

Based on the obtained 7 prognostic genes and their respective coefficients, OC patients were divided into high‐risk group and low‐risk group. To better understand the impact of various clinical features on OC risk, we analyzed the associations between clinical features and different‐risk ovarian cancers. The heatmap depicted in Figure 5A illustrated the correlation between risk groups and clinical information, primarily encompassing stage, age, and grade. The risk scores presented in Figure 5B, along with the consistent findings from both the TCGA‐OC dataset and GSE140082 dataset, indicated that high‐risk patients exhibited elevated mortality rates accompanied by up‐regulated expression levels of prognostic genes *ARID1B*, *HDAC1*, and *INO80*. These genes might serve as potential risk factors for OC development. Conversely, *ATRX*, *CHRAC, MBD2*, and *SS18* were found to be down‐regulated with increasing risk scores, suggesting a possible protective effect. The KM curve analysis was conducted on both the TCGA‐OC dataset and GSE140082 dataset, revealing a significant increase in the mortality rate among patients in the high‐risk group (Figure 5C). Additionally, UMAP and tSNE methods were employed to explore the classification ability of our risk grouping approach, yielding expected outcomes (Figure 5D,E). Overall, this risk model can better identify patients at high risk for OC, so that early intervention can be achieved to reduce the risk of disease progression.

### 3.5 Construction of a Prognostic Nomogram

**FIGURE 6:**
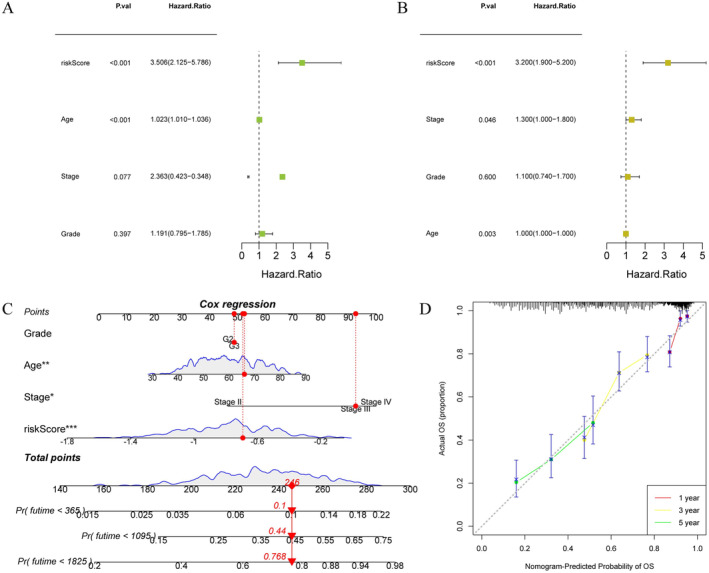
Construction of a nomogram (A) Univariate cox forest map of clinical features; (B) Multivariate cox forest map of clinical features; (C) Clinical prognosis histogram; (D) Calibration curves of the nomogram to predict 1, 3, and 5‐year survival rates.

To achieve a more accurate and comprehensive risk assessment of OC patients, we incorporated both risk scores and clinical characteristics into a nomogram for analysis. Univariate Cox regression analysis revealed that risk score and age were associated with survival in OC patients (Figure 6A). Multivariate Cox regression analysis demonstrated that stage, age, and risk score were independent predictors of OS in OC patients (Figure 6B). In sum, these findings highlighted the independent prognostic value of risk score characteristics in OC patients. When constructing the prognostic nomogram, we selected a representative sample from the OC patient cohort for evaluation, which exhibited a risk score of 245. The prediction results demonstrated an increasing trend in the patients' mortality risks at intervals of 1, 3, and 5 years over time (Figure 6C). As shown in Figure 6D, the calibration curves of 1, 3, and 5 years almost overlap with the diagonal, proving that our model had a high prediction accuracy. In summary, the nomogram we constructed has high accuracy and can provide a personalized prognosis assessment for each OC patient.

### 3.6 Differential Gene Enrichment Analysis Between Risk Groups

**FIGURE 7:**
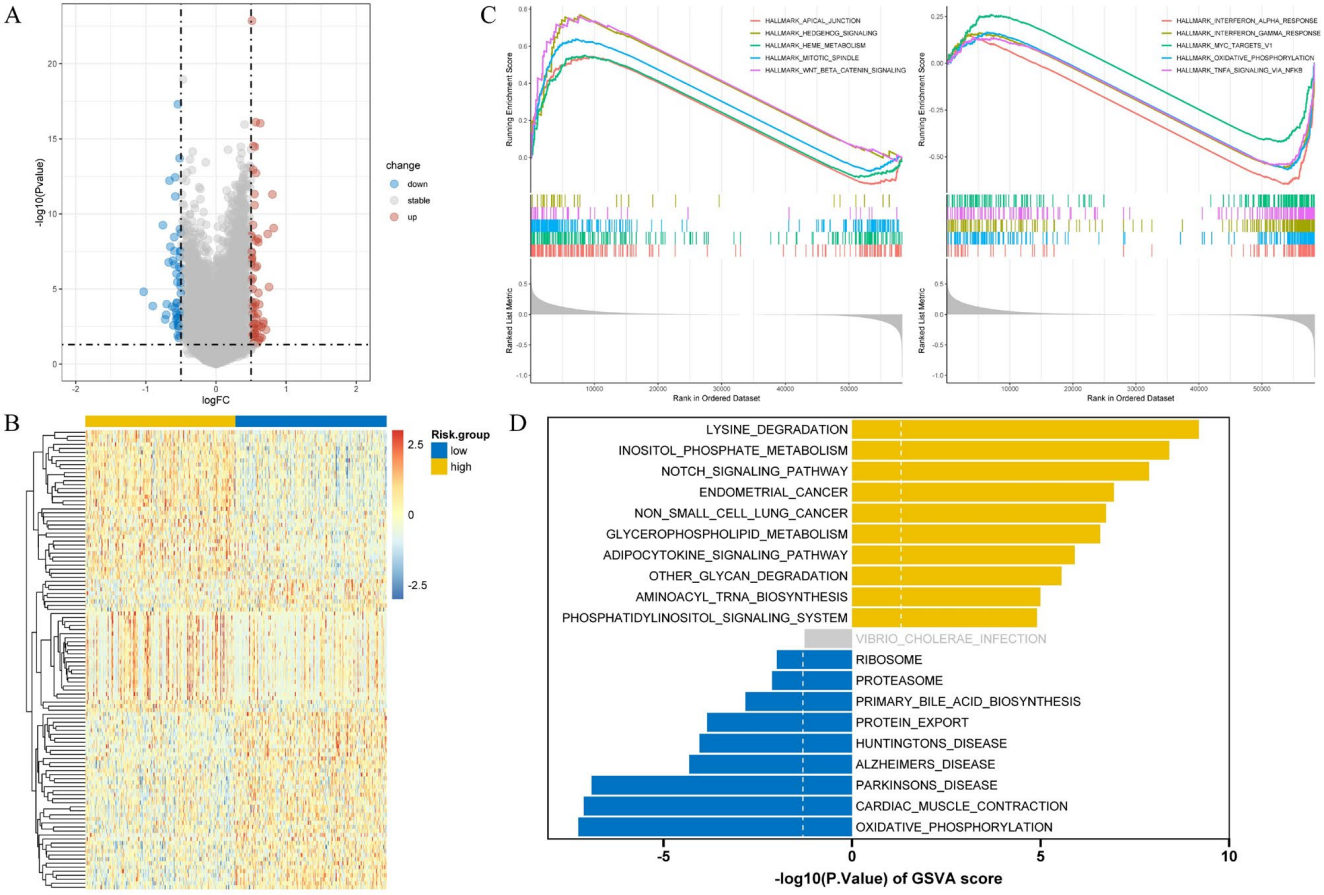
Difference enrichment analysis of high and low risk groups (A) Volcano plot of DEGs in high and low risk groups; (B) Heatmap of DEGs in high and low risk groups; (C) GSEA‐hallmark enrichment analysis; (D) GSVA analyzed the differential KEGG pathway. GSEA, gene set enrichment analysis; GSVA, gene set variation analysis.

In order to further understand the pathological mechanism of OC at the molecular level in different risk groups, we further performed pathway enrichment analysis. By conducting differential gene analysis on high‐risk and low‐risk groups, we identified a total of 114 genes with differential expression patterns, including 52 down‐regulated genes and 62 up‐regulated genes (Figure 7A,B). GSEA revealed the inhibition of pathways such as oxidative phosphorylation and interferon‐α response in the high‐risk group, while cancer‐related pathways including spindle mitosis and the hedgehog signaling pathway were activated (Figure 7C). Furthermore, GSVA demonstrated the presence of both activated and inhibited pathways in the high‐risk group, encompassing the notch signaling pathway, proteasome, and phosphatidylinositol signaling system (Figure 7D).

### 3.7 Immune Cell Infiltration Analysis in Risk Groups

**FIGURE 8:**
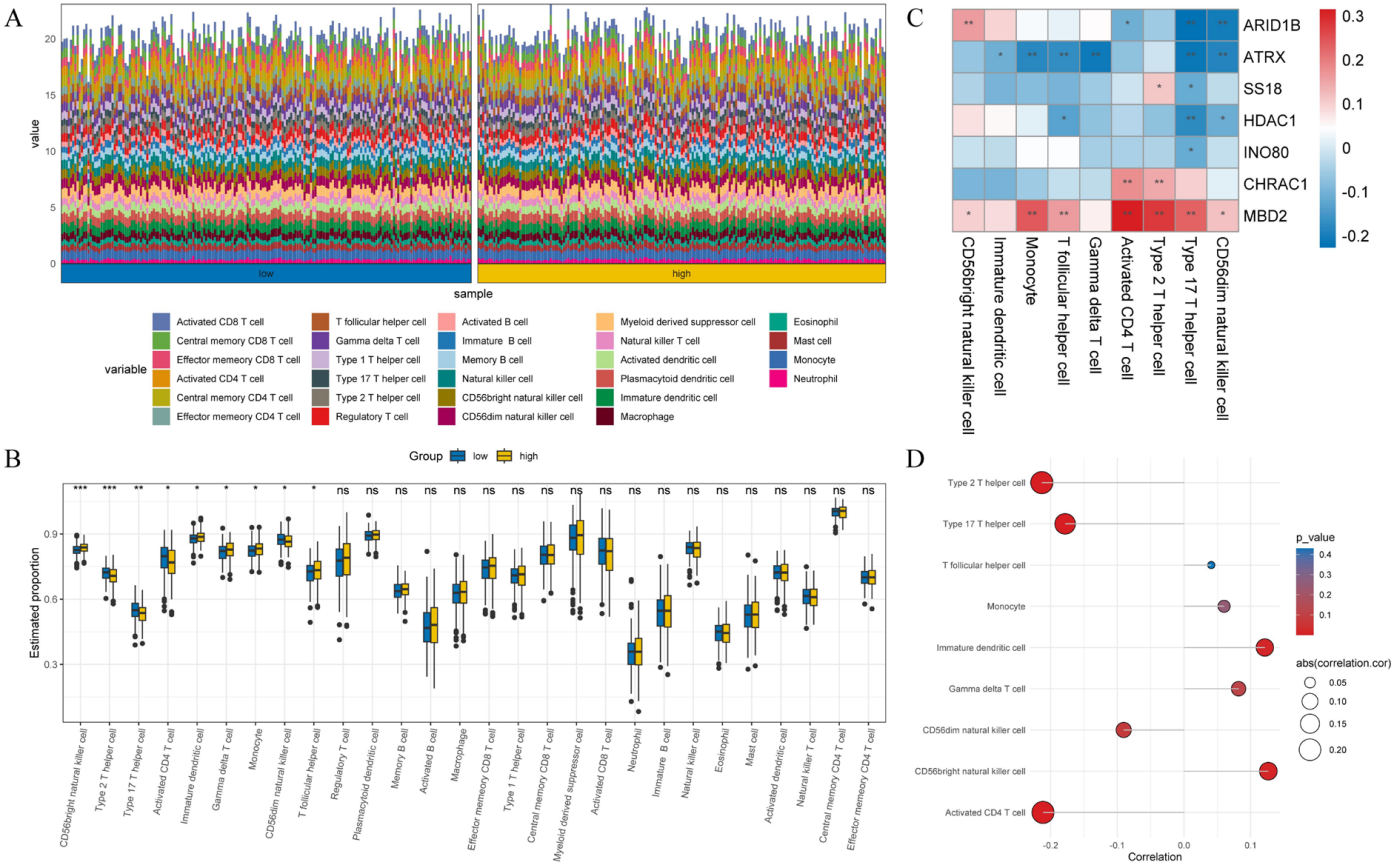
Analysis of immune infiltration (A) Histograms of cell infiltration differences in high and low‐risk groups; (B) Box plots of cell infiltration differences among high and low‐risk groups; (C) Heat map of immune cell infiltration and prognostic genes; (D) Lollipop chart in risk score with cell infiltration.

**FIGURE 9:**
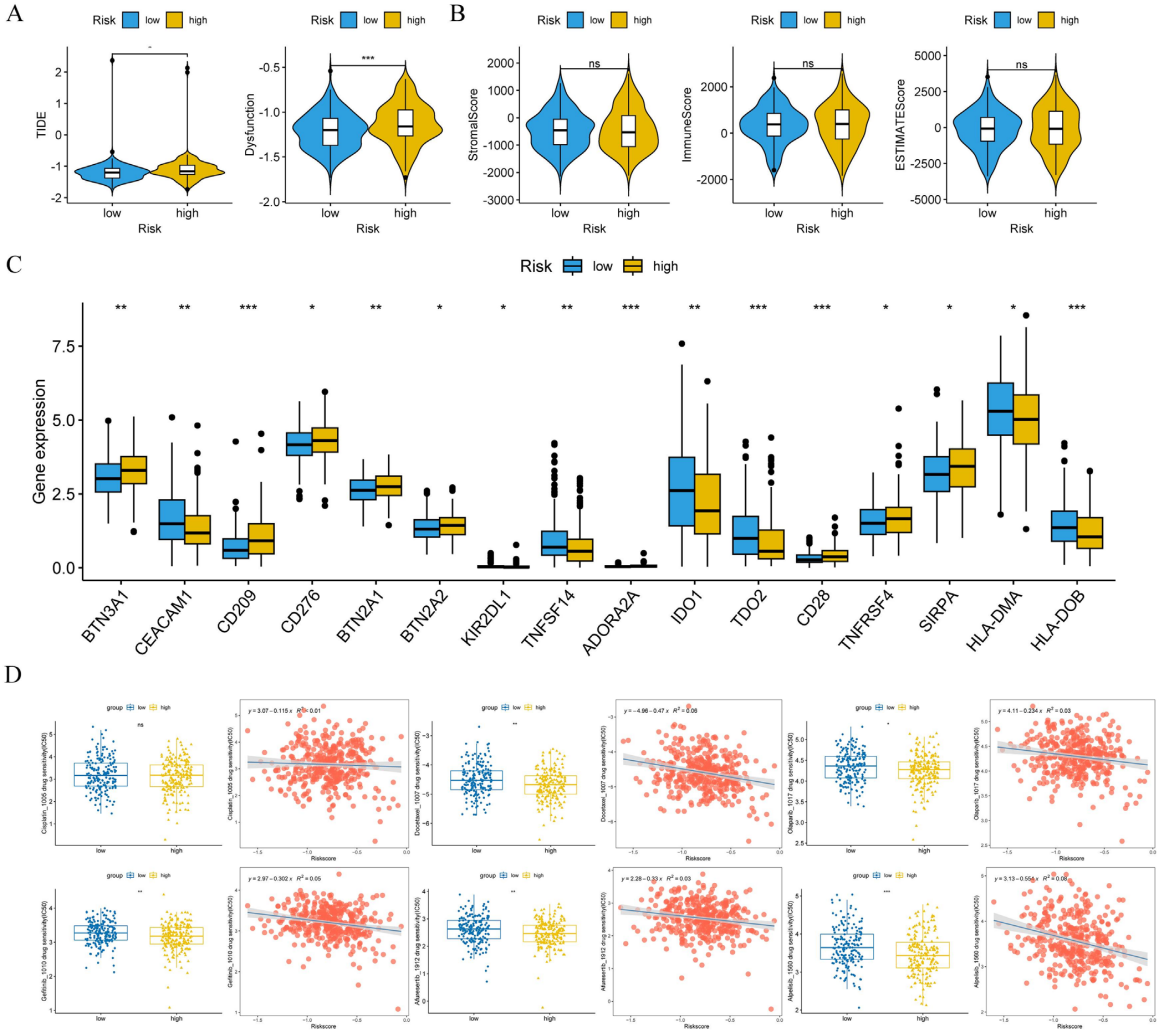
Immunotherapy response in high‐and low‐risk groups and IC_50_ analysis of drug sensitivity. (A) difference analysis; (B) ESTIMATE score difference analysis; (C) immune checkpoint analysis; (D) IC_50_ analysis of drug sensitivity. *p < 0.05; **p < 0.01; ***p < 0.001; ****p < 0.0001. ns, not significant; TIDE, tumor immune dysfunction and exclusion.

Since the immunotherapy for OC has not achieved the expected effect, we performed immunoinfiltration analysis on OC patients with different risk groups to provide a new breakthrough for personalized treatment of OC. Figure 8A showed the infiltration levels of 28 immune cells in the high‐and low‐risk groups for OC patients (Figure 8A). Subsequently, box plots were used to identify immune cells with significant differences in the high‐risk group, including type 2 T helper cells and CD56dim natural killer cells (Figure 8B). Furthermore, we investigated the correlation between the expression level of prognostic genes and immune infiltrating cells in the tumor microenvironment. The results demonstrated that the expression of *MBD2* was positively correlated with the majority of immune cells (Figure 8C). To better understand the relationship between immune cell infiltration and OC prognosis, we performed correlation analyses of risk scores and differential immune cells. The results showed that the risk score exhibited a negative correlation with type 2 T cells and activated CD4 T cells (*p* < 0.05, |cor| > 0.5), while it showed a positive correlation with immature dendritic cells and CD56 bright natural killer cells (*p* < 0.05, |cor| > 0.5) (Figure 8D). Through TIDE analysis, we observed that patients in the high‐risk group showed higher scores in TIDE score and dysfunction, indicating a more significant occurrence of the tumor immune exclusion phenomenon and potentially decreased effectiveness of the immune system against tumor cells (Figure 9A). Additionally, we found no significant differences in stromalscore, immunescore, and estimatescore between high‐and low‐risk groups (Figure 9B). Importantly, the expression levels of immune checkpoint molecules differed significantly between high‐and low‐risk groups, including *IDO1*, *CD209*, and *HLA‐DO8*, et al. (Figure 9C). In conclusion, immunoinfiltration analysis of high‐and low‐risk OC patients helps us understand the role of the tumor immune microenvironment in different risk patients.

### 3.8 Drug Sensitivity Analysis

In order to reveal the difference in drug response in OC patients with different risk groups and provide a theoretical basis for individualized treatment, drug sensitivity analysis was conducted. We evaluated the sensitivity of 179 drugs for each sample, including chemotherapy and molecular targeted agents. The results showed that docetaxel, olaparib, gefitinib, afuresertib, and alpelisib had lower IC_50_ values in the high‐risk group compared to low‐risk group patients, suggesting that high‐risk patients may have heightened responsiveness to conventional chemotherapeutic agents (Figure 9D). It also provides valuable guidance for clinical drug selection.

### 3.9 Experimental Verification of Prognostic Gene Expression Levels

**FIGURE 10:**
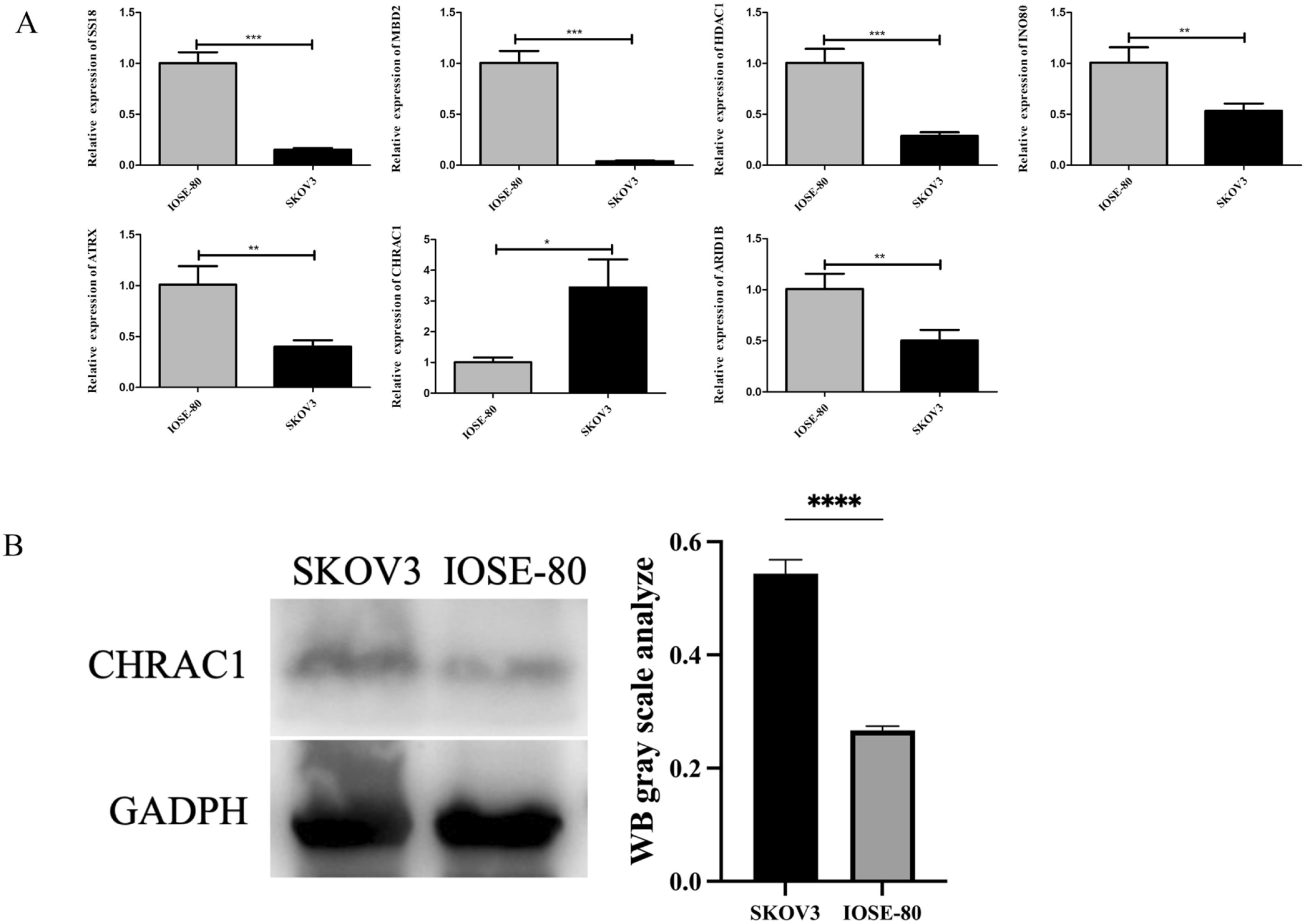
The results of qRT‐PCR and Western blotting. (A) The results of qRT‐PCR ns, not significant; *p < 0.05; **p < 0.01; ***p < 0.001. (B) The results of Western blotting in *CHRAC1*. The molecular weight of GAPDH is 36 kDa, and the molecular weight of CHARC1 is 15 kDa. ****p < 0.0001. The experiment was conducted with biological replicates (*N* = 3). qRT‐PCR, quantitative real‐time polymerase chain reaction.

In this study, qRT‐PCR was used to analyze the differential expressions of *ARID1B*, *ATRX*, *CHRAC1*, *HDAC1*, *INO80*, *MBD2*, and *SS18* in the OC cell line SKOV3 and the human normal ovarian epithelial cell line IOSE‐80. As shown in Figure 10A, the expressions of *SS18*, *MBD2*, *INO80*, *HDAC1*, *CHRAC1*, *ATRX*, and *ARID1B* in SKOV3 cells and IOSE‐80 cells were significantly different (*p* < 0.05), and the expression of *CHRAC1* in SKOV3 cells was significantly increased. After conducting the qRT‐PCR experiment evaluation, to further demonstrate the high expression of CHRAC1 protein in SKOV3 cells, western blotting was performed. The molecular weight of GAPDH is 36 kDa, and the molecular weight of CHARC1 is 15 kDa. The findings revealed a higher expression level of *CHRAC1* in SKOV3 cells compared to IOSE‐80 cells, which aligns with our qRT‐PCR results (Figure 10B).

## 4 Discussion

OC remains the leading cause of mortality among gynecological cancers. Despite extensive research over the years, suitable prognostic genes with diagnostic sensitivity and specificity, and effective screening methods, are still lacking. The pathogenesis of OC remains poorly understood, with suspected involvement of hormonal, genetic, and environmental factors [30, 31]. There existed four conserved families of ATP‐dependent chromatin remodelers in mammals: chromodomain helicase DNA binding (CHD), imitation switch (ISWI), inositol requiring 80 (INO80), and switch/sucrose non‐fermenting (SWI/SNF) [9]. Genes frequently mutated in OC encode proteins involved in chromatin remodeling complexes (CRCs), which play crucial roles in transcription, DNA repair, and the dynamic regulation of chromatin accessibility, supporting cellular physiological processes [10]. Approximately 25% of human cancers exhibit mutations in at least one of 29 genes encoding SWI/SNF proteins [32]. Among these, ARID1A (also known as BAF250a, B120, C1orf4, Osa1) is the most frequently altered gene. ARID1A mutations are particularly prevalent in gynecologic cancers (detected in 10%–60% of ovarian and endometrial carcinoma cases) [33], especially in premalignant gynecological lesions of endometrioid origin [34]. Although existing studies support an association between OC and chromatin remodeling, there have been few studies of prognostic genes associated with chromatin remodeling in OC. Based on this direction, we conducted bioinformatics analysis and ultimately identified 7 prognostic genes: *ARID1B*, *ATRX*, *CHRAC1*, *HDAC1*, *INO80*, *MBD2*, and *SS18*. These genes are associated with chemotherapy resistance and survival rates in OC, primarily enriched in the ATP‐dependent chromatin remodeling pathway, ATP‐dependent chromatin remodeler activity, and notch signaling pathway.

ARID1B exists in the SWI/SNF chromatin remodeling complex in an exclusive manner with ARID1A and has been identified as a major mutated gene in various cancers in recent years. In a prognostic study by Beihe Wang on bladder cancer [35] and Yan Cui's research [36] on triple‐negative breast cancer, ARID1B was associated with poor prognosis. In a multi‐institutional cohort study [37] of 23 cases of dedifferentiated and undifferentiated ovarian carcinomas (DDOC/UDOC), 18 out of 22 cases with interpretable immunohistochemical results showed loss of expression of core SWI/SNF proteins, with 10 cases exhibiting substantial loss of ARID1A and ARID1B. In a previous study [38], the expression level of ARID1B was generally positively correlated with progression‐free survival, which was consistent with our findings in this study. The study by Xinfeng Liu et al. reported [39] that high expression of HDAC1 was associated with poor prognosis in OC, and that resistance to chemotherapeutic drugs was related to overexpression of HDAC1 in OC cells. BCCIP (BRCA2‐and cdkn1a‐interacting protein) serves as a critical cofactor for BRCA2 in tumor suppression. Low expression of BCCIP had been observed in various primary tumor tissues with clinical diagnoses, including OC, renal cell carcinoma, and colorectal cancer. Jiaming Su et al. reported [40] that knockdown of INO80 or YY1 can significantly inhibit BCCIP enrichment, thereby weakening the protective effect on tumor suppression and promoting tumor progression. ATRX was also a prominent factor in OC prognosis research, having been reported as a recurrently mutated driver gene in OC by Jianxiong Li [41]. Our study findings indicated that expression level of ATRX was negatively correlated with poor prognosis, which was consistent with Katherine Foster's research [42], where ATRX mutations were associated with shorter progression‐free survival. However, Ben Davidson's study [43] suggested that the expression level of ATRX was not related to clinicopathological parameters or survival. In terms of drug resistance, Hongbo Yang found [44] that the expression levels of PART1 and CHRAC1 were increased in cisplatin (DDP)‐resistant OC cell lines. Ieva Vaicekauskaitė et al. discovered amplifications of ACTL6A, CHRAC1, and RSF1 in high‐grade serous OC [10]. In a study conducted by Wangang Gong et al. [45], it was found that 63.5% of 73 tissue samples from high‐grade serous ovarian cancer (HGSOC) exhibited positive expression of MBD2, while the expression level of MBD2 was undetectable in all 16 normal tissue samples (100%). Furthermore, compared to platinum‐sensitive cases, MBD2 expression was significantly higher in platinum‐resistant cases. Additionally, high expression of MBD2 was negatively correlated with recurrence‐free survival. These findings aligned with our conclusion that MBD2 was associated with high resistance, supporting the robustness of our analysis. Several studies [46] have reported the role of SS18‐SSX fusion proteins in activating repressor factors in synovial sarcoma, but there are currently no relevant reports on their involvement in OC.

This study comprehensively investigated the prognostic characteristics of CRRGs in OC through bioinformatics analysis, aiming to discover novel biomarkers and therapeutic targets. By integrating data from TCGA, GTEx, and GEO databases, we successfully identified a panel of DECRRGs and further pinpointed 7 prognostic genes: *ARID1B*, *ATRX*, *CHRAC1*, *HDAC1*, *INO80*, *MBD2*, and *SS18*. The expression patterns of these genes were significantly associated with patient prognosis, providing a critical basis for risk assessment in OC. Based on these 7 prognostic genes, we constructed a risk prediction model that classified patients into high‐risk and low‐risk groups through risk score calculation. Notably, patients in the high‐risk group exhibited poorer prognosis and enhanced sensitivity to chemotherapy drugs, suggesting their potential role in modulating chemotherapy response in OC. Additionally, immune infiltration analysis uncovered distinct immune microenvironments between risk groups, offering new insights into OC immune evasion mechanisms. Experimental validations using qRT‐PCR and Western blotting techniques confirmed the specific expression patterns of these prognostic genes in OC tissues, aligning with our bioinformatics predictions and reinforcing the reliability of our model. Functional enrichment analyses revealed that these genes are primarily involved in transcriptional dysregulation and cell cycle progression, shedding light on the pathogenesis and progression of OC. However, the limitations of this study lie in the fact that the findings were based on data analysis from public databases, necessitating validation through the collection of clinical samples. This study only analyzed the specific expression of prognostic genes in SKOV3 cells, and there was a lack of studies in other cell lines or animal models. Additionally, the mechanisms of prognostic genes require investigation in different subtypes of OC through basic experiments, and their prognostic value needs further validation in clinical settings. This also sets the direction for our future research.

## 5 Conclusions

In conclusion, this study presented a CRRG‐based prognostic model that empowers risk stratification and personalized treatment in OC. By elucidating the roles and mechanisms of these prognostic genes and pathways, our findings pave the way for the development of novel targeted therapies and optimization of patient treatment strategies.

## Author Contributions

**Guansheng Chen:** conceptualization, data curation, formal analysis, investigation, methodology, project administration, resources, writing – original draft, writing – review and editing, software. **Wenjing Li:** conceptualization, formal analysis, investigation, writing – review and editing, software. **Jiayi Guo:** data curation, formal analysis, investigation, methodology. **Lingyu Liu:** formal analysis, investigation, methodology, writing – original draft, writing – review and editing. **Yongjun Wang:** conceptualization, data curation, methodology, project administration, resources, supervision, writing – review and editing.

## Conflicts of Interest

The authors declare no conflicts of interest.

## Supporting information


Table S1. A total of 117 chromatin remodeling‐related genes (CRRGs).

Table S2. A total of 57 differentially expressed chromatin remodeling‐related genes (DECRRGs).

**Table S3.** Kyoto Encyclopedia of Genes and Genomes (KEGG) enrichment analysis of DECRRGs.

**Table S4.** Gene Ontology (GO) enrichment analysis of DECRRGs.

Table S5. Changes in expression levels of 7 prognostic genes compared to control.

## Data Availability

The TCGA‐OC dataset was downloaded from the TCGA database (https://portal.gdc.cancer.gov/repository). The GSE140082 dataset was downloaded from the GEO database (https://www.ncbi.nlm.nih.gov/geo). CRRGs were obtained from the GeneCards database (https://www.genecards.org).
