# Supplementary material for: Development and Validation of Prognostic Characteristics Associated With Chromatin Remodeling‐Related Genes in Ovarian Cancer
Source: Cancer Med. 2025 Feb 11;14(3):e70634. doi: 10.1002/cam4.70634 (PMC11811884; doi:10.1002/cam4.70634)
Supplement: Supplementary file 1 — Table S1. A total of 117 chromatin remodeling‐related genes (CRRGs). [file CAM4-14-e70634-s001.docx]

Table S1. 117 Chromatin remodeling-related genes (CRRGs)

| H2AC18 | ARID2 | ACIN1 |
| --- | --- | --- |
| BICRAL | HLTF | CHD1 |
| KTI12 | ZMYND8 | SP1 |
| SPTY2D1 | SMARCA5 | SMARCB1 |
| SPINDOC | RBBP7 | ATRX |
| CRAMP1 | SMARCD1 | TNFRSF11A |
| BICRA | BAZ1B | CTCF |
| H3C14 | BPTF | TNFRSF11B |
| BCL7C | ACTL6B | LBR |
| BCL7A | MTA1 | BRD4 |
| MXRA8 | MTA2 | KAT2B |
| DPF3 | CHD5 | HMGB1 |
| ACTR8 | CHD8 | FLNA |
| H4C1 | H3-3A | KMT2A |
| RSF1 | BRD7 | SMARCA2 |
| CHTOP | SUZ12 | ACTB |
| H4C16 | SMARCC1 | COL1A1 |
| BCL7B | SMARCD2 | SIRT1 |
| CHRAC1 | SMARCAD1 | KAT2A |
| CECR2 | CDT1 | TNFSF11 |
| CHD6 | FLII | HDAC1 |
| CHAF1A | MACROH2A1 | PARP1 |
| INO80 | CHD2 | KAT5 |
| H3C1 | CBX5 | SMARCA4 |
| CHD9 | CBX3 | HDAC2 |
| SS18L1 | GATAD2B | EP300 |
| SUPT16H | RBBP4 | TP53 |
| SS18 | SMARCC2 | MMP9 |
| CHAF1B | CHD3 | CTNNB1 |
| BAZ2A | ARID1B | EZH2 |
| BGLAP | RUVBL2 | CREBBP |
| SRCAP | SATB1 | ESR1 |
| BAZ2B | H2AX | SMARCAL1 |
| POLE3 | NCOA1 | CHD1L |
| MBD2 | ARID1A | ERCC6 |
| SMARCA1 | CHD4 | MBD3 |
| SMARCD3 | PBRM1 | SMARCE1 |
| BAZ1A | RUVBL1 | SSRP1 |
| ACTL6A | HELLS | TRIM28 |
