# Supplementary material for: Development and Validation of Prognostic Characteristics Associated With Chromatin Remodeling‐Related Genes in Ovarian Cancer
Source: Cancer Med. 2025 Feb 11;14(3):e70634. doi: 10.1002/cam4.70634 (PMC11811884; doi:10.1002/cam4.70634)
Supplement: Supplementary file 2 — Table S2. A total of 57 differentially expressed chromatin remodeling‐related genes (DECRRGs). [file CAM4-14-e70634-s004.docx]

Table S2. A total of 57 differentially expressed chromatin remodeling-related genes (DECRRGs)

| MXRA8 | CHD9 | SMARCA1 | SMARCD1 | FLII | NCOA1 | TNFRSF11B |
| --- | --- | --- | --- | --- | --- | --- |
| DPF3 | SUPT16H | SMARCD3 | BPTF | CHD2 | PBRM1 | LBR |
| RSF1 | SS18 | ACIN1 | MTA1 | RBBP4 | TRIM28 | KAT2B |
| CHTOP | BAZ2A | SSRP1 | CHD8 | SMARCC2 | SMARCE1 | HMGB1 |
| CHRAC1 | BGLAP | MBD3 | BRD7 | CHD3 | ERCC6 | FLNA |
| CECR2 | BAZ2B | SMARCA5 | SMARCAD1 | ARID1B | CHD1 | KMT2A |
| CHD6 | MBD2 | RBBP7 | CDT1 | SATB1 | ATRX | SMARCA2 |
| INO80 | KAT2A | HDAC1 | KAT5 | HDAC2 | MMP9 | CTNNB1 |
| CREBBP |  |  |  |  |  |  |
