# Supplementary material for: Development and Validation of Prognostic Characteristics Associated With Chromatin Remodeling‐Related Genes in Ovarian Cancer
Source: Cancer Med. 2025 Feb 11;14(3):e70634. doi: 10.1002/cam4.70634 (PMC11811884; doi:10.1002/cam4.70634)
Supplement: Supplementary file 5 — Table S5. Changes in expression levels of 7 prognostic genes compared to control. [file CAM4-14-e70634-s002.docx]

Table S3. Changes in expression levels of 7 prognostic genes compared to control

|  | logFC | AveExpr | t | P.Value | adj.P.Val | B | change |
| --- | --- | --- | --- | --- | --- | --- | --- |
| ARID1B | -1.565898763 | 2.92061659879362 | -26.83722542 | 1.4209353493993e-96 | 1.45621930628399e-95 | 208.881884062698 | down |
| ATRX | -1.861629637 | 2.50041410974067 | -37.83671069 | 3.88987558996373e-144 | 7.44721708376753e-143 | 318.490072919418 | down |
| CHRAC1 | 1.27431119991354 | 4.52389330591608 | 18.1601635516714 | 4.11664583239578e-56 | 2.52157103992185e-55 | 115.724081804678 | up |
| HDAC1 | -1.138663499 | 5.14788603707597 | -18.9039418 | 1.44433939656677e-59 | 9.29848805945713e-59 | 123.671989828082 | down |
| INO80 | -1.282038829 | 2.51675943945407 | -25.27728276 | 2.2067588887219e-89 | 2.06054506448531e-88 | 192.316381181746 | down |
| MBD2 | -1.436687076 | 3.83286235913438 | -24.53331615 | 6.30541800475712e-86 | 5.63537590587574e-85 | 184.356269028522 | down |
| SS18 | -1.940166127 | 3.89957346814703 | -28.69345781 | 5.17617323062389e-105 | 5.93411288225096e-104 | 228.323830989057 | down |
